# Community Capacity Building for HIV and Addiction Service Integration: An Intervention Trial in Vietnam

**DOI:** 10.1007/s10461-021-03363-0

**Published:** 2021-07-06

**Authors:** Li Li, Chunqing Lin, Li-Jung Liang, Diep Bich Nguyen, Loc Quang Pham, Tuan Anh Le, Tuan Anh Nguyen

**Affiliations:** 1 Semel Institute for Neuroscience and Human Behavior—Center for Community Health, University of California, 10920 Wilshire Blvd., Suite 350, Los Angeles, CA 90024, USA; 2 National Institute of Hygiene and Epidemiology, Hanoi, Vietnam

**Keywords:** Community health worker, HIV, Addiction treatment, Capacity building, Vietnam

## Abstract

Scientific findings and policy guidelines recommend integrating HIV and drug addiction prevention and care into community-based settings. Systematic capacity-building efforts are warranted to provide technical support for community health workers and improve their confidence in the integrated service provision. An intervention trial was conducted between 2018 and 2019 with 120 community health workers (CHW) from 60 communes in Vietnam’s four provinces. The 60 intervention CHW received in-person training to enhance their HIV/addiction-related service knowledge and skills. Online support groups were established between trained CHW and local HIV and addiction specialists. The intervention outcomes were assessed using mixed-effects regression models with the data collected at baseline and every 3 months for 1 year. Adjusted analyses showed that intervention CHW reported a significant increase in the interaction with other treatment providers than the control group at 6 months and remained at the 12-month follow-up. The difference in the improvement of confidence in HIV/addiction-related service delivery between the intervention and control groups was significant at 6-month but became insignificant at the 12-month. Male CHW were more confident in providing services than female CHW at baseline, and gender differences in the changing patterns were observed over time. This capacity-building intervention demonstrated promising outcomes on CHW inter-agency collaborations and confidence in service delivery. Gender divides in healthcare professionals should be attended to in future studies.

## Resumen

Los hallazgos científicos y los protocolos recomiendan integrar la prevención del VIH y la adicción a las drogas en entornos comunitarios. Se implementan esfuerzos sistemáticos de creación de capacidad para brindar apoyo técnico a los trabajadores de salud comunitarios y mejorar su confianza en la prestación de servicios integrados. Se realizó una prueba de intervención entre 2018 y 2019 con 120 trabajadores de salud comunitarios (TSC) de 60 comunas en las cuatro provincias de Vietnam. Los 60 TSC de intervención recibieron capacitación en persona para mejorar sus conocimientos y habilidades de servicios relacionados con VIH / adicción. Se establecieron grupos de apoyo en línea entre TSC capacitados y especialistas locales en VIH y adicciones. Los resultados de la intervención se evaluaron mediante modelos de regresión de efectos mixtos con los datos recopilados al inicio del estudio y cada tres meses durante un año. Los análisis ajustados mostraron que la intervención CHW informó un aumento significativo en la interacción con proveedores de tratamiento diferentes al grupo de control a los seis meses que se mantuvo en el seguimiento a los 12 meses. La diferencia en el aumento de la confianza en la prestación de servicios relacionados con VIH / adicción entre los grupos de intervención y control fue significativa a los 6 meses, pero se volvió insignificante a los 12 meses. Los TSC masculinos tenían más confianza en la prestación de servicios que las femeninas al inicio del estudio, y se observaron diferencias de género en el cambio de patrones a lo largo del tiempo. Esta intervención de creación de capacidad demostró resultados prometedores en las colaboraciones interinstitucionales de los TSC y la confianza en la prestación de servicios. Las brechas de género en los profesionales de la salud deben ser atendidas en estudios futuros.

## Introduction

Due to the high burden of HIV and substance use comorbidities, researchers and physicians are increasingly examining primary care-based approaches to address the comprehensive health needs of HIV-affected populations and populations who use drugs [1-3]. Community health systems are in the perfect position to coordinate community resources, provide culturally appropriate HIV and addiction prevention, and ensure convenient linkage to care [4, 5]. Literature has documented the efficacy of community-based HIV and substance use services in reduced risky behaviors, expanded service coverage, increased patient retention and adherence, and improved health outcomes [6, 7]. Over 30 years ago, the US CDC issued guidelines to provide integrated substance use services in primary care settings [8]. More recently, the World Health Organization also published an operation manual to guide the expansion of HIV testing and prevention, the delivery of accessible treatment, and the provision of sustained patient-oriented care in the community [9].

Despite the scientific findings and policy guidelines, there have been multiple barriers to implementing HIV and drug-related services in the community [3, 10, 11]. Community-based health systems are often resource-constrained, and the providers there are not adequately equipped with knowledge and skills; therefore, they lack the confidence to render HIV/addiction services [12, 13]. Resistance from community health providers is due to stigma towards marginalized populations and the topic’s perceived sensitivity [14]. Community health providers urgently need up-to-date training on evidence-based clinical practice and professional networks of support to conduct clinical work in the area of HIV and harm reduction [15]. However, systematic capacity-building interventions to enhance community health providers’ confidence and capacity in administering HIV and drug-related clinic tasks are currently underdeveloped.

This study was conducted in Vietnam, a country experiencing a concentrated HIV epidemic among people who use drugs [16, 17]. Vietnam has an existing commune health system that forms the grassroots public health network to provide essential preventive and treatment services in the community [18]. The system is currently being deployed to provide universal HIV testing, treatment, and harm reduction services [19]. However, commune health workers (CHW) in Vietnam still hold misconceptions about HIV and drug use and thus perceive various challenges to providing related services [20]. Our team designed and conducted an intervention trial to enhance the capacity of CHW in HIV and drug-related service delivery. The intervention addressed CHW’s technical difficulties in providing HIV/addiction-related services, and at the same time, established a network for them to seek support from HIV and addiction specialists.

This paper presents the intervention outcomes among CHW, focusing on their collaborations with other health agencies to deliver health services. Furthermore, we assessed whether the CHW in the intervention group exhibited a higher level of confidence in HIV/addiction-related service provision than those in the control group. Finally, we explored whether the intervention effect on service confidence differed by CHW’s characteristics. Gender differences were our specific foci because gender-related stereotypes and expectations are widespread and can impact both provider-provider and provider-patient interaction, as well as the quality of health services [21, 22].

## Methods

### Study Design

The cluster randomized controlled trial was conducted in Bac Giang, Hai Duong, Nam Dinh, and Nghe An provinces in Vietnam from March 2018 to December 2019. Sixty commune health centers (CHC) were selected and pair-matched based on the number of patients that the commune serves and the commune’s location. After baseline assessment, each paired CHC was randomized into intervention or control conditions, and two CHW working in each participating CHC were invited to participate in the study. The Institutional Review Board of the participating agencies approved the study protocol. The trial was registered in the ClinicalTrials.gov protocol registration system (NCT03293355).

### Study Participants

The study participants were CHW, including doctors, assistant doctors, nurses, midwives, or pharmacists. Eligible participants were those who (1) were aged 18 or above, and (2) regularly provided health services to community residents in the study CHC. The eligibility was evaluated by trained research staff using a standard script. A total of 120 CHW (60 in the intervention group and 60 in the control group) were recruited from 60 CHC. Participants were informed of the study purpose, procedures, and voluntary participation and signed the informed consent document.

### Intervention Description

The intervention aimed to train and prepare CHW to provide HIV and addiction-related services. The contents focused on enhancing collaboration with HIV and addiction treatment providers and learning tools to provide optimal care for patients living with HIV and/or patients who use drugs. The intervention included three different formats: in-person training sessions, virtual group discussions, and in-person reunion sessions, which were implemented throughout 12 months of the study.

The intervention started with two in-person training sessions, and each session comprised up to 10 CHW. The first session was about learning practical communication skills to improve CHW’s capacity to engage patients who use drugs and patients with HIV receiving care in the community. The content focused on (1) roles, responsibilities, and importance of CHW, (2) challenges of working with people living with HIV and/or patients who use drugs, (3) the stages of behavioral change theory, and (4) practical communication skills and tools. The second session was to strengthen CHW and HIV/addiction specialists’ collaboration to improve communications between CHW and patients. This session also served the purpose of preparing the virtual provider network. CHW were trained to use Facebook groups to communicate with other CHW and health specialists. Also, CHW were encouraged to communicate with patients who use drugs and patients living with HIV more frequently via texting messenger.

At the end of the second session, a Facebook group, including CHW and HIV/addiction specialists in each province, was formed. The platform was where service providers from different disciplines could communicate with each other and exchange professional opinions. For example, service providers could post clinical knowledge, share training resources and opportunities, discuss patient cases, and seek consultation and references. Each group had its own group rules that were agreed upon by all group members to ensure a welcoming group environment, the relevance of discussion topics, and the confidentiality of providers and their patients. In-person reunion sessions were offered once every month during the first 3 months and every 3 months thereafter. The reunion sessions focused on identifying challenges associated with providing HIV/addiction-related services and the corresponding solutions. There were interactive activities, such as prize-giving to the most active participants on the Facebook group, strengthening the group members’ in-person relationships, and engaging them in future online discussions. All participants attended the in-person training sessions and online discussions. More than 84% of the participants attended the reunion sessions.

CHW in the control group received a one-time lecture on HIV- and drug-related topics taught by local health educators after the baseline assessment.

### Data Collection

**Fig. 1:**
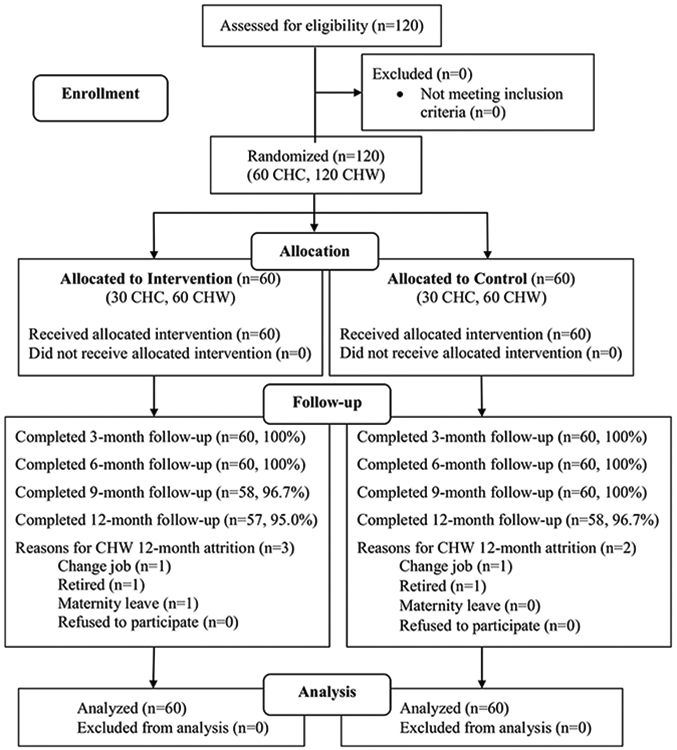
CONSORT for the Cluster Randomized Controlled Trial in Vietnam. *CHC* commune health centers, *CHW* commune health workers

CHW surveys were conducted at baseline and once every 3 months until 12 months. The surveys were administered using an audio computer-assisted self-interview method (ACASI). The computer database was programed to maximize the participants’ privacy and minimize data missing or invalid values. The collection procedures were done in private rooms located in the participating clinics. The CHW read the assessment questions on the computer screen and directly entered their responses into the computer database. A trained staff was available to address technical issues and answer questions during the survey. Each assessment took approximately 40–60 min. Participants received 200,000 VND (approximately 10 USD) as an incentive for each assessment. Figure 1 presents the flow of study participants in the trial.

### Measures

*Confidence in providing HIV/addiction-related services* was assessed by a 6-item scale previously developed and used in Vietnam [19]. Participants rated their confidence in providing services to people living with HIV and patients who use drugs from different aspects. The areas included getting in touch with patients, providing consultation for patients, engaging patients in different treatment programs, helping patients adhere to treatments, motivating patients to make positive behavior changes, and providing long-term follow-up and care for patients. The scale was pilot tested in the field and revised to ensure cultural appropriateness and language clarity. Each item was rated on a 5-point Likert scale, with 1 representing "not confident at all" and 5 representing "very confident." The higher summed score (score range 6–30) indicates a higher confidence level in providing services (Cronbach’s alpha = 0.90).

*Interaction with providers in other healthcare settings* was developed by this team based on previous research experience and learnings from community health workers in Vietnam. There were eight items included in the scale. Each came with five options, ranging from 1 = "None of the time" to 5 = "All of the time." Sample questions included "Service providers in other healthcare settings discuss work-related issues with you," "Service providers in other healthcare settings share work-related documents with you," "Service providers in other healthcare settings are easy to get in touch with when you need their help," "Maintaining a long-term relationship with service providers in other healthcare settings is important to you," and "The information, support, and collaboration you receive from service providers in other healthcare settings are helpful." The overall score was constructed by summing the scores of all items, which falls in the range from 8 to 40. A higher score reflects better interaction with service providers in other healthcare settings (Cronbach’s alpha=0.91).

In addition, CHW demographic and working-related characteristics, including age, gender, educational attainment, working duration at the current CHC, and HIV-related training, were collected.

### Data Analysis

The sample size of 60 CHW per intervention condition (2 per commune by 30 communes) was powered to examine the intervention effect on the continuous outcome measure. This sample size provided at least 80% power at a 5% level of significance to detect a standardized effect size of 0.54 using a two-sided test, assuming a priori intraclass correlation of 0.03 and an attrition rate of up to 5% at 12-month follow-up.

The CHW’s demographics and worked-related characteristics at baseline were summarized using descriptive statistics. Continuous (e.g., age, education, and time worked at the community health center) and categorical (e.g., gender, medical training, occupation, and other related training) characteristics were compared between the intervention and control at baseline using two-sample *t* tests and Chi-squared tests, respectively.

An intent-to-treat approach was used for all the analyses. A linear mixed-effects regression model was used to assess the intervention effect on the outcome measures: interaction with healthcare providers from other agencies and confidence in providing HIV/addiction-related services. The unadjusted regression model included the following fixed-effects: intervention condition, visit, and two-way intervention-by-visit interaction. The pre-selected characteristics were added to the unadjusted model to assess whether the intervention effects remain after controlling these characteristics. Each model also included two levels of random effects, commune- and participant-level, to account for dependence within communes and correlations between repeated observations for each individual. At each of the 6- and 12-month follow-up visits, each outcome measure’s mean change score (i.e., mean change from baseline) for each condition was estimated. A difference in difference approach was used to estimate the intervention effect on each outcome measure, i.e., the difference in change scores between intervention and control, through model contrasts. A stratified analysis by gender using the regression model described above was conducted to explore whether there was a gender variation in the intervention effects on confidence in providing services among CHW. All statistical analyses were performed using the SAS System version 9.4 [SAS Institute Inc., Cary, NC, USA].

## Results

### Participant Characteristics at Baseline

Table 1 shows the demographics and working-related characteristics of all participants at baseline. The mean age for this sample was 42.3 years old, and the average years of education were around 15 years. The majority of the CHW were female (72.5%). Of 120 CHW, 58.3% were doctors or assistant doctors. Less than half (42.5%) had worked at the current CHC for more than 20 years. Sixty-six CHW (55.0%) had previously received addiction treatment-related training since working at the current CHC, and 93 (77.5%) had received HIV/AIDS training before the baseline survey. No significant differences in any of the above characteristics were observed between the intervention and control groups at baseline.

### Intervention Effects on Outcome Measures

Intervention effects on CHW’s interaction with providers from the other agencies and confidence in service, adjusting for the pre-selected characteristics, are shown in Table 2. At 6-month, the CHW in the intervention group reported an increased level of interaction with providers from other agencies from baseline, whereas those in the control group stayed at a similar level as the baseline (estimated change score from the baseline: 2.04 vs. −0.70, respectively); the difference in change scores between groups was significant (difference ± SE: 2.74 ± 0.94; p = 0.004). The intervention effect on the level of interaction with providers from other agencies waned slightly but remained significant at the 12-month follow-up (2.08 ±0.95; p = 0.029). After controlling for the pre-selected characteristics (adjusted analysis), the intervention effects were similar to the improvement of this outcome at 6- and 12-month follow-up visits (2.83±0.95 and 2.03±0.97, respectively; p-values <0.0.05, Table 2). The estimated mean scores at baseline for CHW’s confidence in service were similar between intervention and control (21.3 vs. 21.8, respectively). At 6-month, the CHW in the intervention group reported a significantly increased level of confidence in service from baseline compared to those in the control group (estimated difference in change scores: 1.64±0.75, p = 0.030). After controlling for the pre-selected characteristics, results were similar (1.81 ± 0.76, p = 0.017; Table 2). The adjusted difference in improvement between the two groups became smaller at the 12-month follow-up visit and did not reach statistical significance (1.33 ± 0.76, p = 0.081; Table 2). Gender was significantly associated with the level of confidence in providing services, where male CHW reported a higher level of confidence in providing services to patients (1.33 ± 0.62, p = 0.033).

### Exploratory Analysis

**Fig. 2:**
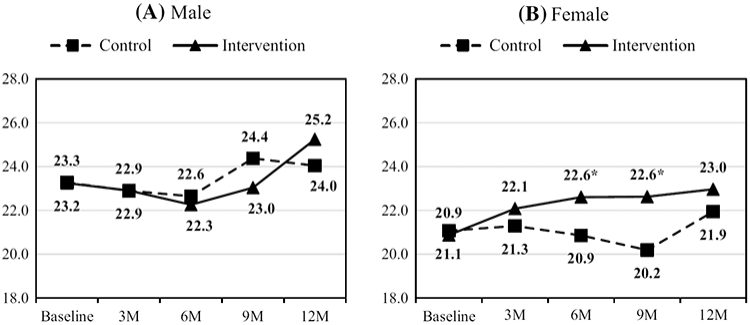
Mean scores of confident in service by intervention over time for **A** Male and **B** Female. Mean scores of confidence levels in service provision were estimated using linear mixed-effects models, stratified by gender. In each figure, the dashed line with a square symbol represents the control group’s means, and the solid line with a triangular symbol represents the intervention group’s estimated means. *p < 0.05

Figure 2 presents the gender-stratified analysis results, i.e., the estimated mean score of confidence in providing services over time by group and gender. At baseline, the mean score for the male CHW was significantly higher than the female CHW (mean score: 23.5 vs. 20.6, p = 0.002). For male CHW (in Fig. 2A), the mean scores for both intervention and control increased slowly over time; however, the difference in change scores between the two groups did not reach statistical significance. For female CHW, the mean score increased steadily over time for the intervention group, whereas that for the control group stayed around or below the baseline level for the first 9 months of the study and then went up slightly at the end of the study. The difference in change scores was significant at 6-month (mean change score: 1.73 vs. −0.21, p = 0.030) and 9-month (1.75 vs. −0.89, p = 0.003), but no longer significant at the end of the study.

## Discussion

The study demonstrated the efficacy of a capacity-building intervention in CHW’s cross-agency collaboration and their confidence level in providing HIV/addiction-related services. The intervention effect on provider interaction was significant at 6-month and remained over the 12-month study period. This outcome might be achieved by integrating both in-person and online components. Both formats were interactive to maximize participants’ attention and engagement. After CHW established the connection with HIV and addiction specialists via face-to-face contact in the initial in-person sessions, they continued to communicate with one another to exchange medical expertise, discuss cases, and refer patients through the virtual online network autonomously and spontaneously. These findings expand upon the similarly encouraging findings of our prior intervention, which focused exclusively on MMT providers and HIV specialists [23]. The uninterrupted online communication explained the sustainability of the intervention outcome in provider interaction at 12 months. We observed interaction on the Facebook groups even after the 12-month study period. Such online training and networking modality are convenient, flexible, and low-cost. It is particularly pertinent to continue medical training in this COVID19 era when physical distancing has become the new normalcy [24].

Through training in practical skills to engage patients who use drugs and patients with HIV in the community, intervention group CHW demonstrated a higher confidence level in providing HIV and drug services at 6-month. However, the intervention effect became insignificant at 12-month. The result recommends continuous effort in future training to better equip CHW with high self-efficacy to incorporate what they have learned into their medical practice. An interesting finding is that female CHW were less confident in providing HIV and addiction-related services at baseline, which is contradictory to previous literature where gender was found to have null associations with health workers’ self-efficacy in the identification and treatment of HIV or substance use disorders in other countries [25, 26]. The gender differences in confidence found in this study should be interpreted in the context of Vietnam, where people living with HIV and people who use drugs are predominantly male, while CHW are mostly female [17, 27]. Male patients would recognize female service providers as women rather than trained professionals with authority [28]. In the provision of comprehensive HIV prevention and care, engaging in conversation about sexual risk is inevitable; however, the longstanding gender inequality in Vietnam constrains female providers’ willingness and comfort level to deliver counseling to reduce male patients’ sexual risk [28, 29]. These gender perspectives would contribute to the difficulties in establishing productive therapeutic relationships between opposite-sex providers and patients [28]. Our intervention benefited female CHW by granting them a higher level of confidence in service provision. We observed a steadily increasing trend in female CHW’s confidence in providing HIV/addiction-related services through the intervention. At the end of the study, female CHW in the intervention group achieved a significantly higher confidence level than the females in the control group; yet it still fell below the male intervention providers’ confidence level. Future CHW capacity-building interventions should incorporate a specially designed component to empower providers to handle embarrassment, discomfort, distrust, or misconceptions that may occur due to provider-patient gender discordance [30].

Strengths of the trial include randomization of matched pairs of clinics to intervention or control conditions and high recruitment and follow-up rates of CHW participants. There are also a few study limitations to be acknowledged. The study was conducted in provinces in Northern Vietnam, where internet services are steadily available. The intervention model and outcomes might not be generalizable to other areas with limited internet connectivity. The outcome measures were self-reported and could suffer from social desirability bias. For example, CHW may over-report their interactions with other providers and confidence to provide services, and accurate records of CHW’s communication frequency and service quality were not documented to validate the CHW’s reports.

## Conclusion

This study’s results demonstrate that participation in a combined in-person and online training strengthened CHW’s inter-agency network and improved their confidence in providing HIV/addiction-related health services. The intervention has implications for training a cadre of community health providers in harm reduction and HIV service provision in Vietnam and worldwide.

## Figures and Tables

**Table 1:** Demographic and working-related characteristics of CHW (N = 120)

	Intervention (n = 60) N (%) or Mean (SD)	Control (n = 60) N (%) or Mean (SD)	p
Age, N (%)^a^			0.579
< 35 years	13 (21.7)	18 (30.0)	
35–44 years	16 (26.7)	14 (23.3)	
> 44 years	31 (51.7)	28 (46.7)	
Mean (SD)	42.7 (8.7)	41.9 (9.4)	
Female^a^	47 (78.3)	40 (66.7)	0.152
Education (years), Mean (SD)^b^	15.2 (2.3)	15.4 (2.1)	0.769
Medical training, N (%)^a^			
Two years or less	37 (61.7)	38 (63.3)	0.899
Three years	7 (11.7)	8 (13.3)	
Four years or above	16 (26.7)	14 (23.3)	
Occupation, N (%)^a^			
Doctor	13 (21.7)	9 (15.0)	0.464
Assistant doctor	26 (43.3)	22 (36.7)	
Others (nurse, midwife, pharmacist, etc.)	21 (35.0)	29 (48.3)	
Time at the commune health center, N (%)^a^			
< 10 years	15 (25.0)	20 (33.3)	0.408
10–20 years	16 (26.7)	18 (30.0)	
> 20 years	29 (48.3)	22 (36.7)	
Mean (SD)	17.9 (9.4)	15.5 (8.8)	
Addiction treatment-related training, N (%)^a^	34 (56.7)	32 (53.3)	0.714
HIV/AIDS-related training, N (%)^a^	44 (73.3)	49 (81.7)	0.274

a Chi-squared test was conducted

b Two-independent sample t-test was conducted

**Table 2:** Results from mixed-effects regressions on CHW outcome measures

	Interaction with providers		Confidence in service	
	Estimate (SE)	p	Estimate (SE)	p
Intervention effect^a^				
6 Months	2.83 (0.95)	0.003	1.81 (0.76)	0.017
12 Months	2.03 (0.97)	0.036	1.33 (0.76)	0.081
Covariate				
Age				
< 35 years	−1.32 (1.34)	0.326	−0.33 (1.08)	0.763
35–44 years	−1.02 (0.96)	0.290	−0.39 (0.78)	0.614
> 44 years	REF		REF	
Female	−0.09 (0.76)	0.910	−1.33 (0.62)	0.033
Medical training				
2 years or less	−1.98 (1.94)	0.308	0.19 (1.51)	0.817
3 years	−1.67 (1.55)	0.280	−3.42 (1.22)	0.945
4 years or above	REF		REF	
Occupation				
Doctor	REF		REF	
Assistant doctor	−0.06 (0.92)	0.952	−0.14 (0.73)	0.852
Others	−1.06 (0.94)	0.261	−0.18 (0.76)	0.817
Time worked at community health center				
< 10 years	2.62 (1.40)	0.062	−0.89 (1.04)	0.395
10–20 years	1.98 (1.23)	0.107	1.98 (0.94)	0.035
> 20 years	REF		REF	
HIV/AIDS-related training, N (%)	0.35 (0.73)	0.632	−0.19 (0.60)	0.751

a Intervention effect estimated difference in change scores from baseline between intervention and control
